# Pulmonary Artery Intimal Sarcoma

**DOI:** 10.5334/jbsr.3688

**Published:** 2024-11-13

**Authors:** Karel Mercken, Vincent Sneyers, Emanuele Di Dedda

**Affiliations:** 1 Department Radiology, UZ Leuven, campus Gasthuisberg, Leuven, Belgium

**Keywords:** Pulmonary artery sarcoma, acute angles, wall eclipsing sign

## Teaching Point

An intravascular mass with acute angles, wall eclipsing sign, and absence of vascular tapering, together with negative thrombotic history and risk factors, strongly suggest pulmonary intima sarcoma over pulmonary embolism.

## Case

A 70‑year‑old female patient presented with dyspnea on effort and left‑sided pleuritic chest pain. Medical history includes breast carcinoma and pancreatic intraepithelial neoplasia. On the basis of initial external computed tomography (CT) findings, anticoagulation therapy with a direct oral anticoagulant was initiated. Scintigraphy demonstrated severely limited perfusion of the left lung with preserved ventilation (ventilation [V]/perfusion [Q] mismatch). D‑dimer levels were mildly elevated (553 µg/L: normal value ≤500).

**Figure 1:**
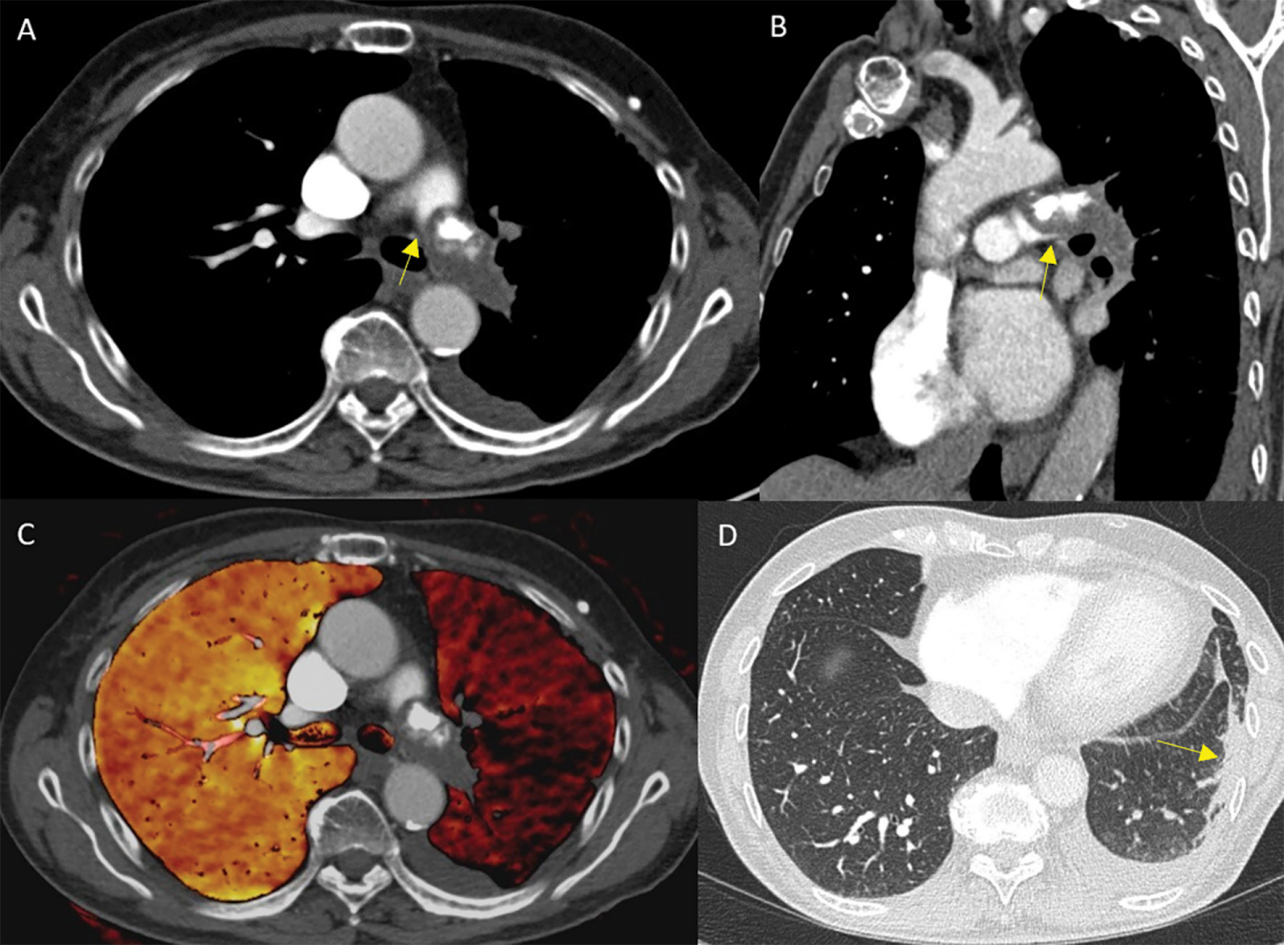
Pulmonary artery intimal sarcoma in the left pulmonary artery presenting as an occlusive, partially calcified mass with broad intimal attachement **(A‑B)** and acute angles (**A‑B**, arrows). The Dual Energy map shows a globally reduced perfusion of the left lung **(C)**. A band‑like irregular consolidation in the left lower lobe is apparent, compatible with sequelae of pulmonary infarction with or without a component of atelectasis **(D)**.

Subsequent CT imaging revealed a persisting partially calcified occlusive mass within the left pulmonary artery, without clear opacification of the pulmonary arteries in the left lung (Figure 1A–B) with globally reduced perfusion of the left lung (Figure 1C). The differential diagnosis included chronic calcified pulmonary embolus or a tumoral mass originating from the pulmonary artery. A band‑like irregular consolidation in the left lower lobe was apparent, and was suspected to be sequelae of pulmonary infarction with or without a component of atelectasis (Figure 1D, arrow). Additionally, mild pleural effusion was present on the left side.

**Figure 2:**
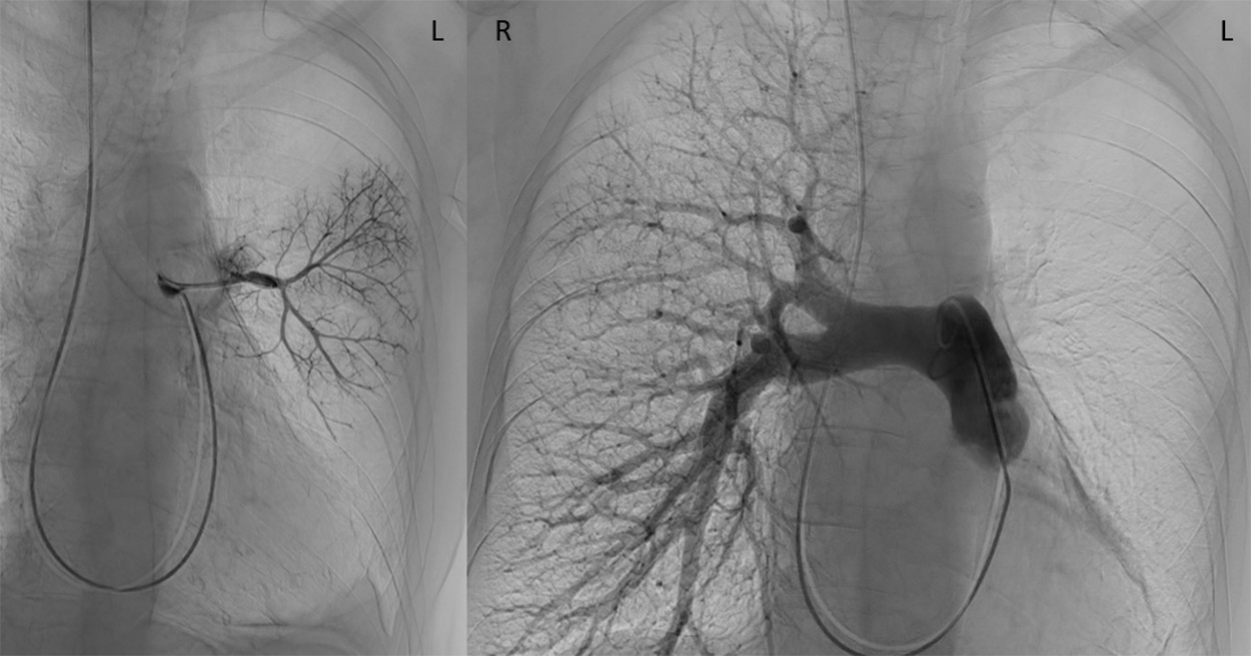
Selective digital subtraction angiography (DSA) of the pulmonary arteries showing filiform patency of the superior lingular branch and an anteromedial basal branch. No opacification of the left lung vasculature was observed upon injection into the pulmonary trunk.

Digital subtraction angiography (DSA) of the pulmonary arteries revealed filiform patency of the superior lingular branch and an anteromedial basal branch. However, no flow to the left lung was observed upon injection into the pulmonary trunk (Figure 2).

**Figure 3:**
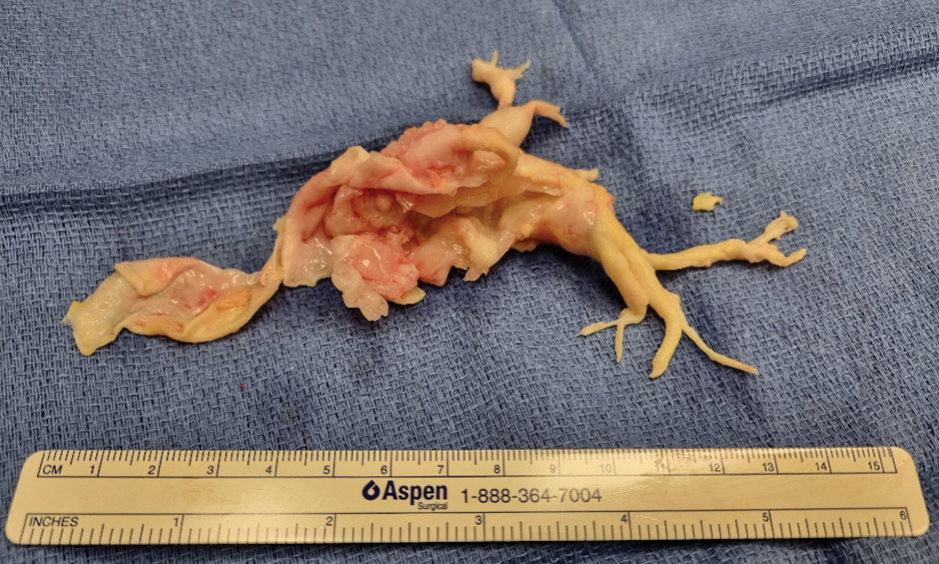
Pulmonary endarterectomy resection piece revealing a nearly complete obstructive mass with pathological intimal involvement extending to the lobular bifurcation.

Pulmonary endarterectomy was performed, revealing a nearly complete obstructive mass with pathological intimal involvement extending to the lobular bifurcation (Figure 3). Pathology findings were suggestive of intimal sarcoma with pronounced chondroblastic differentiation. Fluorescence in situ hybridization (FISH) supported the diagnosis, showing positive MDM2 amplification in 62% of nuclei and a heterogeneous pattern with strong focal platelet‑derived growth factor receptor alpha (PDGFRA) amplification. Wedge resection of the left lower lobe exhibited ischemic necrosis consistent with pulmonary infarction.

## Comment

Pulmonary intimal sarcoma (PAS) presents unique imaging characteristics upon contrast‑enhanced chest CT and CT pulmonary angiography, aiding in its differentiation from pulmonary embolism (PE). PAS often manifests as a convex mass with smooth, lobulated, or irregular contours within the central pulmonary artery, with broad attachment to the intima (Figure 1B). Unlike chronic PE, PAS also forms acute angles with the vessel wall, indicating a distinct growth pattern (Figure 1A–B, arrows). Vascular expansion with antegrade or retrograde intraluminal growth is typical for PAS. The “wall eclipsing” sign, observed as (nearly) occlusive filling defects that extend across vessel walls and propagating toward the right ventricular outflow tract, is characteristic of PAS, preceding extravascular invasion (Figure 1A–B). Contrast enhancement within the mass (on dual phase pulmonary CT) and extravascular extension support a neoplastic diagnosis. Dual‑energy CT can potentially differentiate between tumor and thrombus through material separation techniques. Indicators favoring PAS over chronic (calcifying) PE are the presence of acute lesion margins, the absence of vascular tapering and webs, and positive findings on positron emission tomography (PET) imaging [1].
